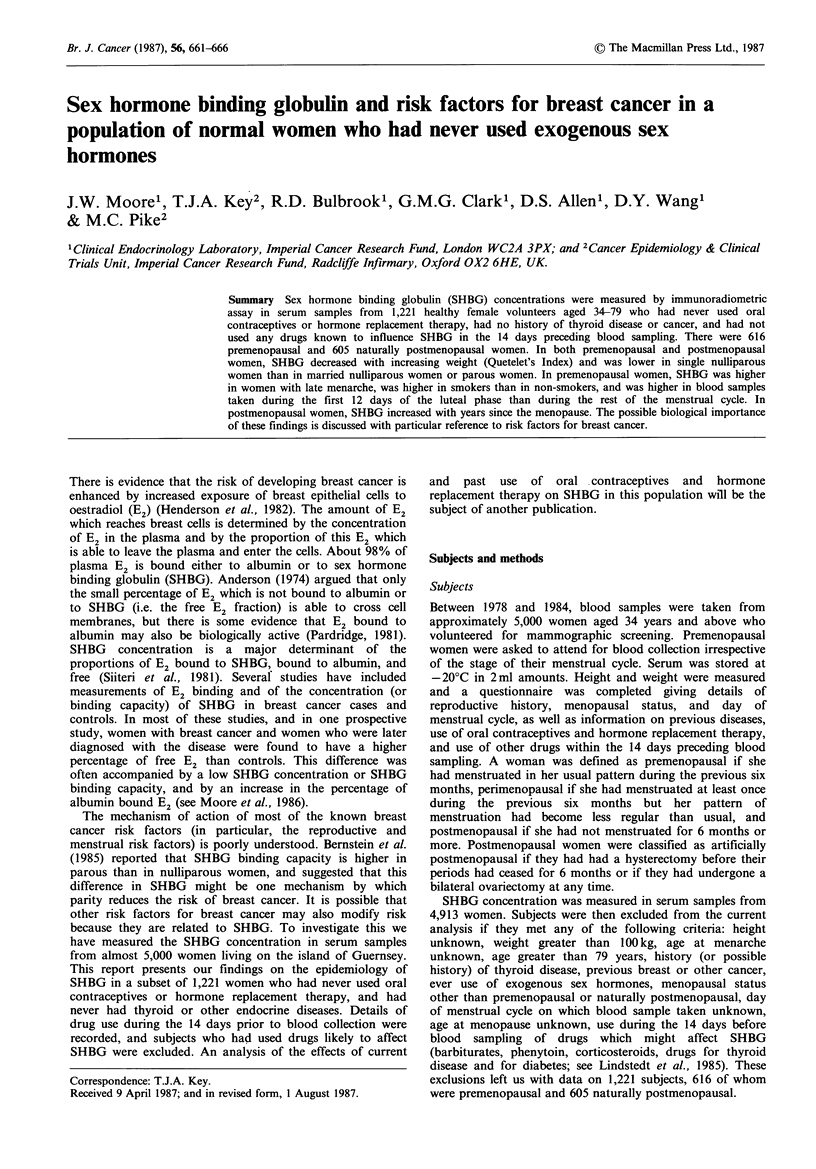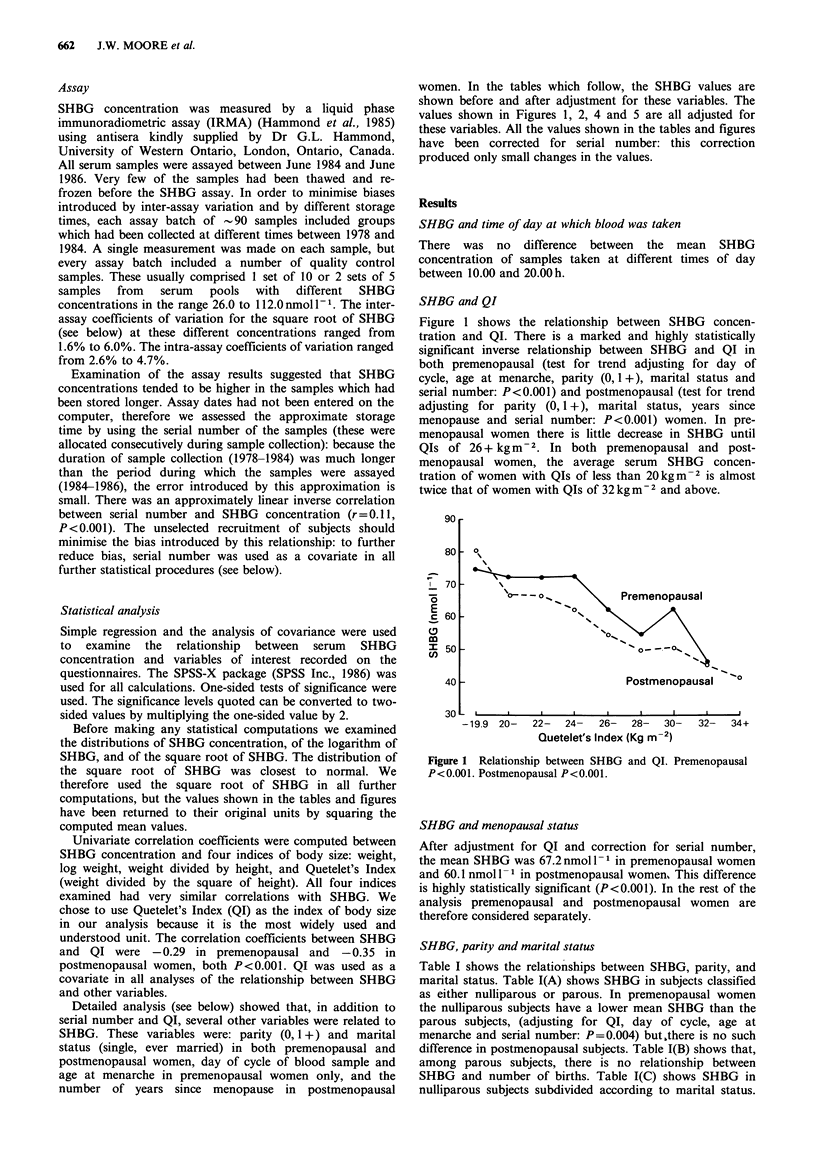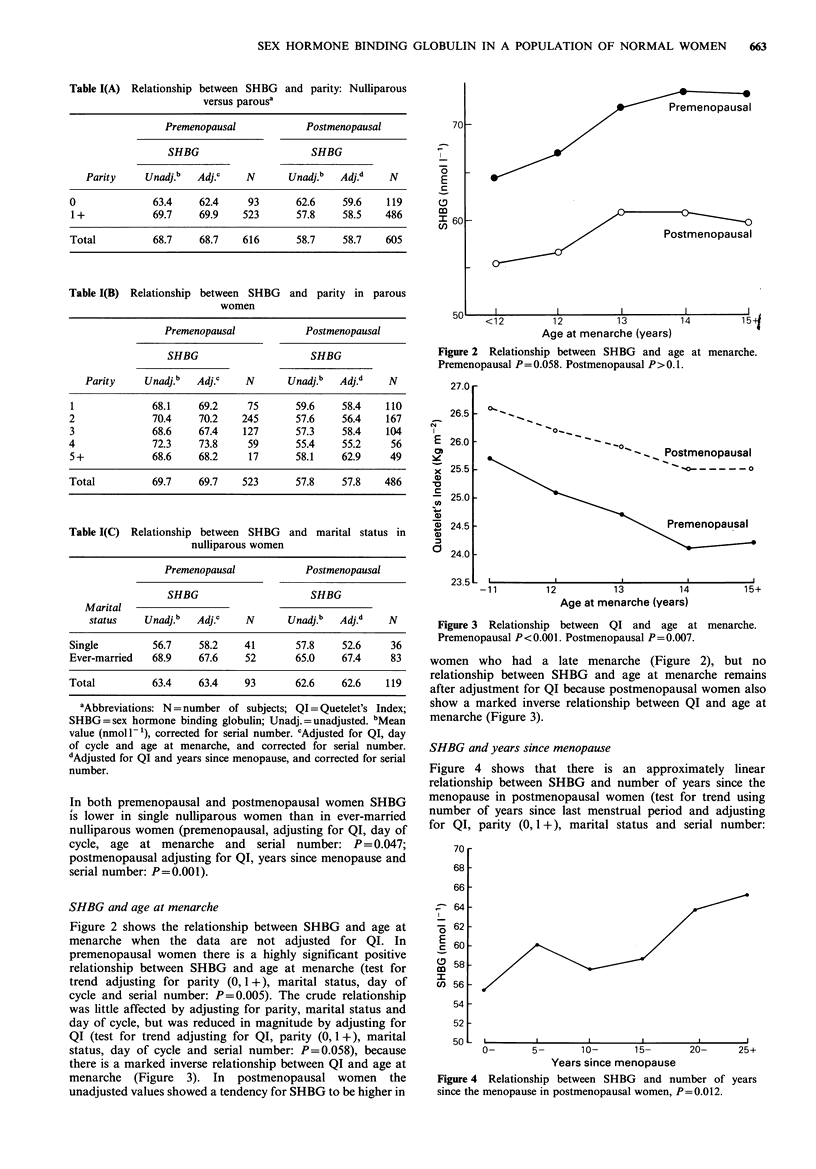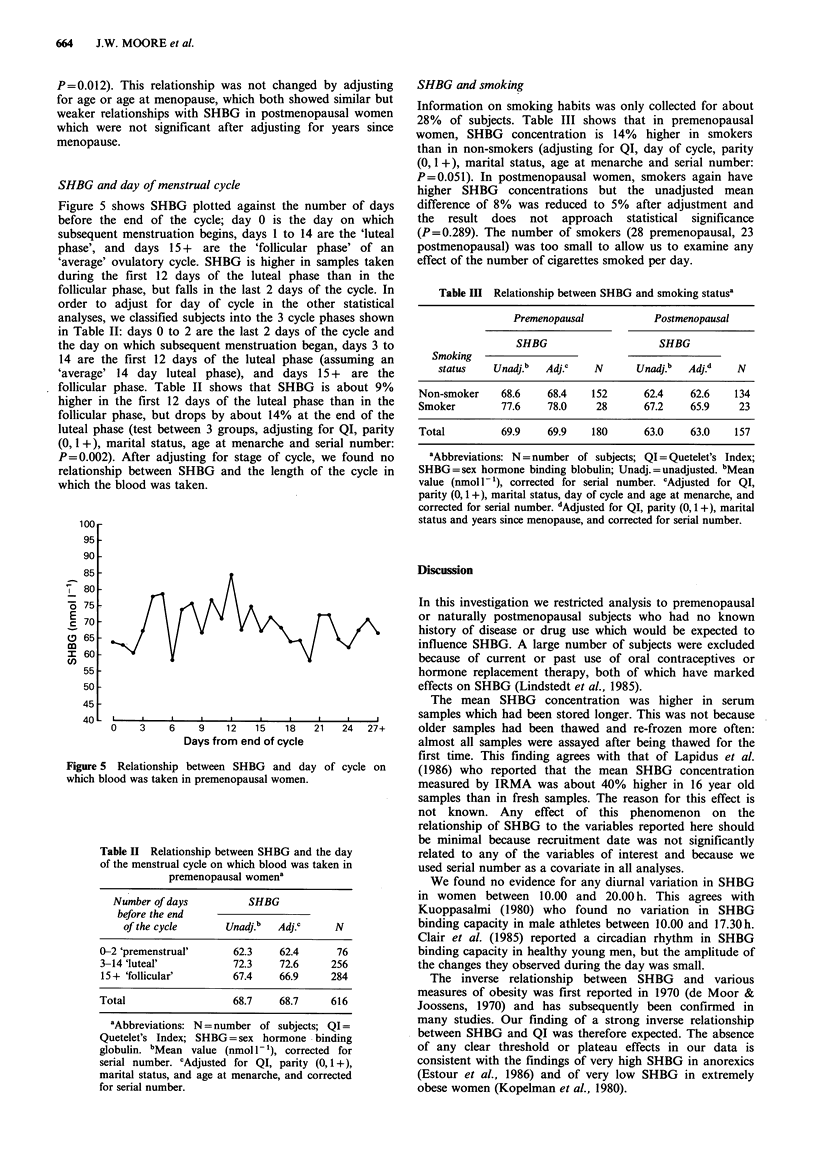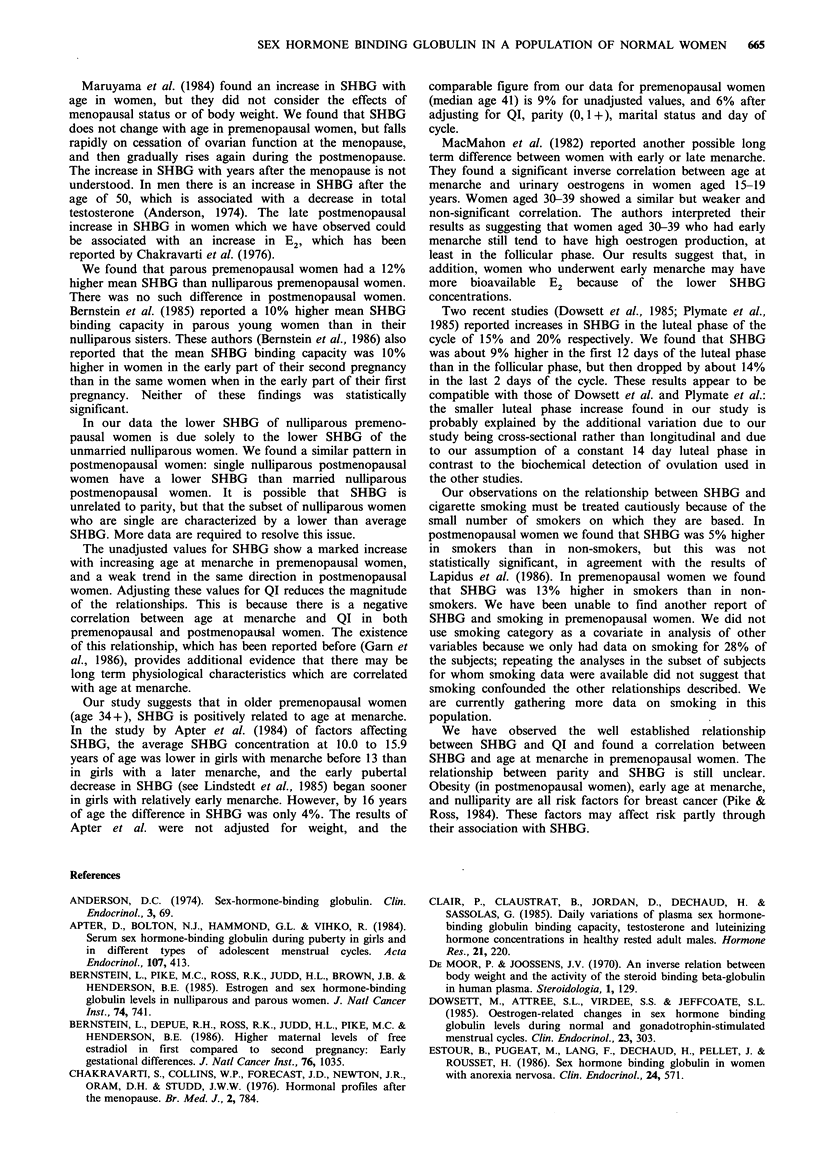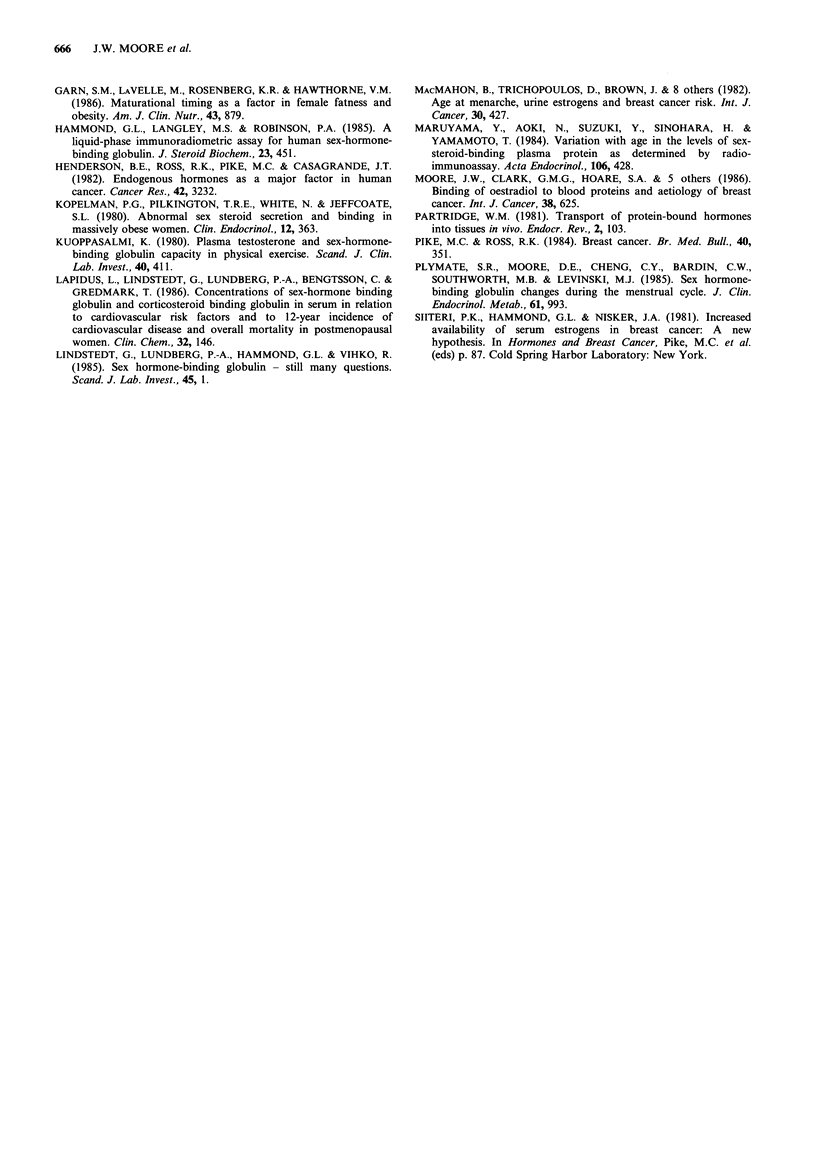# Sex hormone binding globulin and risk factors for breast cancer in a population of normal women who had never used exogenous sex hormones.

**DOI:** 10.1038/bjc.1987.262

**Published:** 1987-11

**Authors:** J. W. Moore, T. J. Key, R. D. Bulbrook, G. M. Clark, D. S. Allen, D. Y. Wang, M. C. Pike

**Affiliations:** Clinical Endocrinology Laboratory, Imperial Cancer Research Fund, London, UK.

## Abstract

Sex hormone binding globulin (SHBG) concentrations were measured by immunoradiometric assay in serum samples from 1,221 healthy female volunteers aged 34-79 who had never used oral contraceptives or hormone replacement therapy, had no history of thyroid disease or cancer, and had not used any drugs known to influence SHBG in the 14 days preceding blood sampling. There were 616 premenopausal and 605 naturally postmenopausal women. In both premenopausal and postmenopausal women, SHBG decreased with increasing weight (Quetelt's Index) and was lower in single nulliparous women than in married nulliparous women or parous women. In premenopausal women, SHBG was higher in women with late menarche, was higher in smokers than in non-smokers, and was higher in blood samples taken during the first 12 days of the luteal phase than during the rest of the menstrual cycle. In postmenopausal women, SHBG increased with years since the menopause. The possible biological importance of these findings is discussed with particular reference to risk factors for breast cancer.


*Br. J. Cancer* (1987), **56**, 661–666 © The Macmillan Press Ltd., 1987

### Sex hormone binding globulin and risk factors for breast cancer in a population of normal women who had never used exogenous sex hormones

J.W. Moore[1], T.J.A. Key[2], R.D. Bulbrook[1], G.M.G. Clark[1], D.S. Allen[1], D.Y. Wang[1] & M.C. Pike[2]

[1]*Clinical Endocrinology Laboratory, Imperial Cancer Research Fund, London WC2A 3PX; and* [2]*Cancer Epidemiology & Clinical Trials Unit, Imperial Cancer Research Fund, Radcliffe Infirmary, Oxford OX2 6HE, UK.*

**Summary** Sex hormone binding globulin (SHBG) concentrations were measured by immunoradiometric assay in serum samples from 1,221 healthy female volunteers aged 34–79 who had never used oral contraceptives or hormone replacement therapy, had no history of thyroid disease or cancer, and had not used any drugs known to influence SHBG in the 14 days preceding blood sampling. There were 616 premenopausal and 605 naturally postmenopausal women. In both premenopausal and postmenopausal women, SHBG decreased with increasing weight (Quetelet's Index) and was lower in single nulliparous women than in married nulliparous women or parous women. In premenopausal women, SHBG was higher in women with late menarche, was higher in smokers than in non-smokers, and was higher in blood samples taken during the first 12 days of the luteal phase than during the rest of the menstrual cycle. In postmenopausal women, SHBG increased with years since the menopause. The possible biological importance of these findings is discussed with particular reference to risk factors for breast cancer.

There is evidence that the risk of developing breast cancer is enhanced by increased exposure of breast epithelial cells to oestradiol ($E_2$) (Henderson *et al.*, 1982). The amount of $E_2$ which reaches breast cells is determined by the concentration of $E_2$ in the plasma and by the proportion of this $E_2$ which is able to leave the plasma and enter the cells. About 98% of plasma $E_2$ is bound either to albumin or to sex hormone binding globulin (SHBG). Anderson (1974) argued that only the small percentage of $E_2$ which is not bound to albumin or to SHBG (i.e. the free $E_2$ fraction) is able to cross cell membranes, but there is some evidence that $E_2$ bound to albumin may also be biologically active (Pardridge, 1981). SHBG concentration is a major determinant of the proportions of $E_2$ bound to SHBG, bound to albumin, and free (Siiteri *et al.*, 1981). Several studies have included measurements of $E_2$ binding and of the concentration (or binding capacity) of SHBG in breast cancer cases and controls. In most of these studies, and in one prospective study, women with breast cancer and women who were later diagnosed with the disease were found to have a higher percentage of free $E_2$ than controls. This difference was often accompanied by a low SHBG concentration or SHBG binding capacity, and by an increase in the percentage of albumin bound $E_2$ (see Moore *et al.*, 1986).

The mechanism of action of most of the known breast cancer risk factors (in particular, the reproductive and menstrual risk factors) is poorly understood. Bernstein *et al.* (1985) reported that SHBG binding capacity is higher in parous than in nulliparous women, and suggested that this difference in SHBG might be one mechanism by which parity reduces the risk of breast cancer. It is possible that other risk factors for breast cancer may also modify risk because they are related to SHBG. To investigate this we have measured the SHBG concentration in serum samples from almost 5,000 women living on the island of Guernsey. This report presents our findings on the epidemiology of SHBG in a subset of 1,221 women who had never used oral contraceptives or hormone replacement therapy, and had never had thyroid or other endocrine diseases. Details of drug use during the 14 days prior to blood collection were recorded, and subjects who had used drugs likely to affect SHBG were excluded. An analysis of the effects of current and past use of oral contraceptives and hormone replacement therapy on SHBG in this population will be the subject of another publication.

Correspondence: T.J.A. Key.
Received 9 April 1987; and in revised form, 1 August 1987.

### Subjects and methods

### *Subjects*

Between 1978 and 1984, blood samples were taken from approximately 5,000 women aged 34 years and above who volunteered for mammographic screening. Premenopausal women were asked to attend for blood collection irrespective of the stage of their menstrual cycle. Serum was stored at −20°C in 2 ml amounts. Height and weight were measured and a questionnaire was completed giving details of reproductive history, menopausal status, and day of menstrual cycle, as well as information on previous diseases, use of oral contraceptives and hormone replacement therapy, and use of other drugs within the 14 days preceding blood sampling. A woman was defined as premenopausal if she had menstruated in her usual pattern during the previous six months, perimenopausal if she had menstruated at least once during the previous six months but her pattern of menstruation had become less regular than usual, and postmenopausal if she had not menstruated for 6 months or more. Postmenopausal women were classified as artificially postmenopausal if they had had a hysterectomy before their periods had ceased for 6 months or if they had undergone a bilateral ovariectomy at any time.

SHBG concentration was measured in serum samples from 4,913 women. Subjects were then excluded from the current analysis if they met any of the following criteria: height unknown, weight greater than 100 kg, age at menarche unknown, age greater than 79 years, history (or possible history) of thyroid disease, previous breast or other cancer, ever use of exogenous sex hormones, menopausal status other than premenopausal or naturally postmenopausal, day of menstrual cycle on which blood sample taken unknown, age at menopause unknown, use during the 14 days before blood sampling of drugs which might affect SHBG (barbiturates, phenytoin, corticosteroids, drugs for thyroid disease and for diabetes; see Lindstedt *et al.*, 1985). These exclusions left us with data on 1,221 subjects, 616 of whom were premenopausal and 605 naturally postmenopausal.

*Assay*

SHBG concentration was measured by a liquid phase immunoradiometric assay (IRMA) (Hammond *et al.*, 1985) using antisera kindly supplied by Dr G.L. Hammond, University of Western Ontario, London, Ontario, Canada. All serum samples were assayed between June 1984 and June 1986. Very few of the samples had been thawed and re-frozen before the SHBG assay. In order to minimise biases introduced by inter-assay variation and by different storage times, each assay batch of ~90 samples included groups which had been collected at different times between 1978 and 1984. A single measurement was made on each sample, but every assay batch included a number of quality control samples. These usually comprised 1 set of 10 or 2 sets of 5 samples from serum pools with different SHBG concentrations in the range 26.0 to 112.0 nmol $l^{-1}$. The inter-assay coefficients of variation for the square root of SHBG (see below) at these different concentrations ranged from 1.6% to 6.0%. The intra-assay coefficients of variation ranged from 2.6% to 4.7%.

Examination of the assay results suggested that SHBG concentrations tended to be higher in the samples which had been stored longer. Assay dates had not been entered on the computer, therefore we assessed the approximate storage time by using the serial number of the samples (these were allocated consecutively during sample collection): because the duration of sample collection (1978–1984) was much longer than the period during which the samples were assayed (1984–1986), the error introduced by this approximation is small. There was an approximately linear inverse correlation between serial number and SHBG concentration ($r=0.11$, $P<0.001$). The unselected recruitment of subjects should minimise the bias introduced by this relationship: to further reduce bias, serial number was used as a covariate in all further statistical procedures (see below).

*Statistical analysis*

Simple regression and the analysis of covariance were used to examine the relationship between serum SHBG concentration and variables of interest recorded on the questionnaires. The SPSS-X package (SPSS Inc., 1986) was used for all calculations. One-sided tests of significance were used. The significance levels quoted can be converted to two-sided values by multiplying the one-sided value by 2.

Before making any statistical computations we examined the distributions of SHBG concentration, of the logarithm of SHBG, and of the square root of SHBG. The distribution of the square root of SHBG was closest to normal. We therefore used the square root of SHBG in all further computations, but the values shown in the tables and figures have been returned to their original units by squaring the computed mean values.

Univariate correlation coefficients were computed between SHBG concentration and four indices of body size: weight, log weight, weight divided by height, and Quetelet's Index (weight divided by the square of height). All four indices examined had very similar correlations with SHBG. We chose to use Quetelet's Index (QI) as the index of body size in our analysis because it is the most widely used and understood unit. The correlation coefficients between SHBG and QI were −0.29 in premenopausal and −0.35 in postmenopausal women, both $P<0.001$. QI was used as a covariate in all analyses of the relationship between SHBG and other variables.

Detailed analysis (see below) showed that, in addition to serial number and QI, several other variables were related to SHBG. These variables were: parity (0, 1+) and marital status (single, ever married) in both premenopausal and postmenopausal women, day of cycle of blood sample and age at menarche in premenopausal women only, and the number of years since menopause in postmenopausal women. In the tables which follow, the SHBG values are shown before and after adjustment for these variables. The values shown in Figures 1, 2, 4 and 5 are all adjusted for these variables. All the values shown in the tables and figures have been corrected for serial number: this correction produced only small changes in the values.

### Results

*SHBG and time of day at which blood was taken*

There was no difference between the mean SHBG concentration of samples taken at different times of day between 10.00 and 20.00 h.

*SHBG and QI*

Figure 1 shows the relationship between SHBG concentration and QI. There is a marked and highly statistically significant inverse relationship between SHBG and QI in both premenopausal (test for trend adjusting for day of cycle, age at menarche, parity (0, 1+), marital status and serial number: $P<0.001$) and postmenopausal (test for trend adjusting for parity (0, 1+), marital status, years since menopause and serial number: $P<0.001$) women. In premenopausal women there is little decrease in SHBG until QIs of 26+ kg $m^{-2}$. In both premenopausal and postmenopausal women, the average serum SHBG concentration of women with QIs of less than 20 kg $m^{-2}$ is almost twice that of women with QIs of 32 kg $m^{-2}$ and above.

**Figure 1** Relationship between SHBG and QI. Premenopausal $P<0.001$. Postmenopausal $P<0.001$.

*SHBG and menopausal status*

After adjustment for QI and correction for serial number, the mean SHBG was 67.2 nmol $l^{-1}$ in premenopausal women and 60.1 nmol $l^{-1}$ in postmenopausal women. This difference is highly statistically significant ($P<0.001$). In the rest of the analysis premenopausal and postmenopausal women are therefore considered separately.

*SHBG, parity and marital status*

Table I shows the relationships between SHBG, parity, and marital status. Table I(A) shows SHBG in subjects classified as either nulliparous or parous. In premenopausal women the nulliparous subjects have a lower mean SHBG than the parous subjects, (adjusting for QI, day of cycle, age at menarche and serial number: $P=0.004$) but there is no such difference in postmenopausal subjects. Table I(B) shows that, among parous subjects, there is no relationship between SHBG and number of births. Table I(C) shows SHBG in nulliparous subjects subdivided according to marital status.

**Table I(A)** Relationship between SHBG and parity: Nulliparous versus parous[a]

| | Premenopausal | | | Postmenopausal | | |
|---|---|---|---|---|---|---|
| | SHBG | | | SHBG | | |
| *Parity* | *Unadj.*[b] | *Adj.*[c] | *N* | *Unadj.*[b] | *Adj.*[d] | *N* |
| 0 | 63.4 | 62.4 | 93 | 62.6 | 59.6 | 119 |
| 1+ | 69.7 | 69.9 | 523 | 57.8 | 58.5 | 486 |
| Total | 68.7 | 68.7 | 616 | 58.7 | 58.7 | 605 |

**Table I(B)** Relationship between SHBG and parity in parous women

| | Premenopausal | | | Postmenopausal | | |
|---|---|---|---|---|---|---|
| | SHBG | | | SHBG | | |
| *Parity* | *Unadj.*[b] | *Adj.*[c] | *N* | *Unadj.*[b] | *Adj.*[d] | *N* |
| 1 | 68.1 | 69.2 | 75 | 59.6 | 58.4 | 110 |
| 2 | 70.4 | 70.2 | 245 | 57.6 | 56.4 | 167 |
| 3 | 68.6 | 67.4 | 127 | 57.3 | 58.4 | 104 |
| 4 | 72.3 | 73.8 | 59 | 55.4 | 55.2 | 56 |
| 5+ | 68.6 | 68.2 | 17 | 58.1 | 62.9 | 49 |
| Total | 69.7 | 69.7 | 523 | 57.8 | 57.8 | 486 |

**Table I(C)** Relationship between SHBG and marital status in nulliparous women

| | Premenopausal | | | Postmenopausal | | |
|---|---|---|---|---|---|---|
| | SHBG | | | SHBG | | |
| *Marital status* | *Unadj.*[b] | *Adj.*[c] | *N* | *Unadj.*[b] | *Adj.*[d] | *N* |
| Single | 56.7 | 58.2 | 41 | 57.8 | 52.6 | 36 |
| Ever-married | 68.9 | 67.6 | 52 | 65.0 | 67.4 | 83 |
| Total | 63.4 | 63.4 | 93 | 62.6 | 62.6 | 119 |

[a]Abbreviations: N = number of subjects; QI = Quetelet's Index; SHBG = sex hormone binding globulin; Unadj. = unadjusted. [b]Mean value ($nmol\,l^{-1}$), corrected for serial number. [c]Adjusted for QI, day of cycle and age at menarche, and corrected for serial number. [d]Adjusted for QI and years since menopause, and corrected for serial number.

In both premenopausal and postmenopausal women SHBG is lower in single nulliparous women than in ever-married nulliparous women (premenopausal, adjusting for QI, day of cycle, age at menarche and serial number: $P = 0.047$; postmenopausal adjusting for QI, years since menopause and serial number: $P = 0.001$).

### *SHBG and age at menarche*

Figure 2 shows the relationship between SHBG and age at menarche when the data are not adjusted for QI. In premenopausal women there is a highly significant positive relationship between SHBG and age at menarche (test for trend adjusting for parity (0, 1+), marital status, day of cycle and serial number: $P = 0.005$). The crude relationship was little affected by adjusting for parity, marital status and day of cycle, but was reduced in magnitude by adjusting for QI (test for trend adjusting for QI, parity (0, 1+), marital status, day of cycle and serial number: $P = 0.058$), because there is a marked inverse relationship between QI and age at menarche (Figure 3). In postmenopausal women the unadjusted values showed a tendency for SHBG to be higher in

**Figure 2** Relationship between SHBG and age at menarche. Premenopausal $P = 0.058$. Postmenopausal $P > 0.1$.

**Figure 3** Relationship between QI and age at menarche. Premenopausal $P < 0.001$. Postmenopausal $P = 0.007$.

women who had a late menarche (Figure 2), but no relationship between SHBG and age at menarche remains after adjustment for QI because postmenopausal women also show a marked inverse relationship between QI and age at menarche (Figure 3).

### *SHBG and years since menopause*

Figure 4 shows that there is an approximately linear relationship between SHBG and number of years since the menopause in postmenopausal women (test for trend using number of years since last menstrual period and adjusting for QI, parity (0, 1+), marital status and serial number:

**Figure 4** Relationship between SHBG and number of years since the menopause in postmenopausal women, $P = 0.012$.

$P=0.012$). This relationship was not changed by adjusting for age or age at menopause, which both showed similar but weaker relationships with SHBG in postmenopausal women which were not significant after adjusting for years since menopause.

*SHBG and day of menstrual cycle*

Figure 5 shows SHBG plotted against the number of days before the end of the cycle; day 0 is the day on which subsequent menstruation begins, days 1 to 14 are the 'luteal phase', and days 15+ are the 'follicular phase' of an 'average' ovulatory cycle. SHBG is higher in samples taken during the first 12 days of the luteal phase than in the follicular phase, but falls in the last 2 days of the cycle. In order to adjust for day of cycle in the other statistical analyses, we classified subjects into the 3 cycle phases shown in Table II: days 0 to 2 are the last 2 days of the cycle and the day on which subsequent menstruation began, days 3 to 14 are the first 12 days of the luteal phase (assuming an 'average' 14 day luteal phase), and days 15+ are the follicular phase. Table II shows that SHBG is about 9% higher in the first 12 days of the luteal phase than in the follicular phase, but drops by about 14% at the end of the luteal phase (test between 3 groups, adjusting for QI, parity (0, 1+), marital status, age at menarche and serial number: $P=0.002$). After adjusting for stage of cycle, we found no relationship between SHBG and the length of the cycle in which the blood was taken.

**Figure 5** Relationship between SHBG and day of cycle on which blood was taken in premenopausal women.

**Table II** Relationship between SHBG and the day of the menstrual cycle on which blood was taken in premenopausal women[a]

| *Number of days before the end of the cycle* | *SHBG* | | |
|---|---|---|---|
| | *Unadj.*[b] | *Adj.*[c] | *N* |
| 0–2 'premenstrual' | 62.3 | 62.4 | 76 |
| 3–14 'luteal' | 72.3 | 72.6 | 256 |
| 15+ 'follicular' | 67.4 | 66.9 | 284 |
| Total | 68.7 | 68.7 | 616 |

[a]Abbreviations: N = number of subjects; QI = Quetelet's Index; SHBG = sex hormone binding globulin. [b]Mean value (nmol $l^{-1}$), corrected for serial number. [c]Adjusted for QI, parity (0, 1+), marital status, and age at menarche, and corrected for serial number.

*SHBG and smoking*

Information on smoking habits was only collected for about 28% of subjects. Table III shows that in premenopausal women, SHBG concentration is 14% higher in smokers than in non-smokers (adjusting for QI, day of cycle, parity (0, 1+), marital status, age at menarche and serial number: $P=0.051$). In postmenopausal women, smokers again have higher SHBG concentrations but the unadjusted mean difference of 8% was reduced to 5% after adjustment and the result does not approach statistical significance ($P=0.289$). The number of smokers (28 premenopausal, 23 postmenopausal) was too small to allow us to examine any effect of the number of cigarettes smoked per day.

**Table III** Relationship between SHBG and smoking status[a]

| *Smoking status* | *Premenopausal* | | | *Postmenopausal* | | |
|---|---|---|---|---|---|---|
| | *SHBG* | | | *SHBG* | | |
| | *Unadj.*[b] | *Adj.*[c] | *N* | *Unadj.*[b] | *Adj.*[d] | *N* |
| Non-smoker | 68.6 | 68.4 | 152 | 62.4 | 62.6 | 134 |
| Smoker | 77.6 | 78.0 | 28 | 67.2 | 65.9 | 23 |
| Total | 69.9 | 69.9 | 180 | 63.0 | 63.0 | 157 |

[a]Abbreviations: N = number of subjects; QI = Quetelet's Index; SHBG = sex hormone binding blobulin; Unadj. = unadjusted. [b]Mean value (nmol $l^{-1}$), corrected for serial number. [c]Adjusted for QI, parity (0, 1+), marital status, day of cycle and age at menarche, and corrected for serial number. [d]Adjusted for QI, parity (0, 1+), marital status and years since menopause, and corrected for serial number.

### Discussion

In this investigation we restricted analysis to premenopausal or naturally postmenopausal subjects who had no known history of disease or drug use which would be expected to influence SHBG. A large number of subjects were excluded because of current or past use of oral contraceptives or hormone replacement therapy, both of which have marked effects on SHBG (Lindstedt *et al.*, 1985).

The mean SHBG concentration was higher in serum samples which had been stored longer. This was not because older samples had been thawed and re-frozen more often: almost all samples were assayed after being thawed for the first time. This finding agrees with that of Lapidus *et al.* (1986) who reported that the mean SHBG concentration measured by IRMA was about 40% higher in 16 year old samples than in fresh samples. The reason for this effect is not known. Any effect of this phenomenon on the relationship of SHBG to the variables reported here should be minimal because recruitment date was not significantly related to any of the variables of interest and because we used serial number as a covariate in all analyses.

We found no evidence for any diurnal variation in SHBG in women between 10.00 and 20.00 h. This agrees with Kuoppasalmi (1980) who found no variation in SHBG binding capacity in male athletes between 10.00 and 17.30 h. Clair *et al.* (1985) reported a circadian rhythm in SHBG binding capacity in healthy young men, but the amplitude of the changes they observed during the day was small.

The inverse relationship between SHBG and various measures of obesity was first reported in 1970 (de Moor & Joossens, 1970) and has subsequently been confirmed in many studies. Our finding of a strong inverse relationship between SHBG and QI was therefore expected. The absence of any clear threshold or plateau effects in our data is consistent with the findings of very high SHBG in anorexics (Estour *et al.*, 1986) and of very low SHBG in extremely obese women (Kopelman *et al.*, 1980).

Maruyama *et al.* (1984) found an increase in SHBG with age in women, but they did not consider the effects of menopausal status or of body weight. We found that SHBG does not change with age in premenopausal women, but falls rapidly on cessation of ovarian function at the menopause, and then gradually rises again during the postmenopause. The increase in SHBG with years after the menopause is not understood. In men there is an increase in SHBG after the age of 50, which is associated with a decrease in total testosterone (Anderson, 1974). The late postmenopausal increase in SHBG in women which we have observed could be associated with an increase in $E_2$, which has been reported by Chakravarti *et al.* (1976).

We found that parous premenopausal women had a 12% higher mean SHBG than nulliparous premenopausal women. There was no such difference in postmenopausal women. Bernstein *et al.* (1985) reported a 10% higher mean SHBG binding capacity in parous young women than in their nulliparous sisters. These authors (Bernstein *et al.*, 1986) also reported that the mean SHBG binding capacity was 10% higher in women in the early part of their second pregnancy than in the same women when in the early part of their first pregnancy. Neither of these findings was statistically significant.

In our data the lower SHBG of nulliparous premenopausal women is due solely to the lower SHBG of the unmarried nulliparous women. We found a similar pattern in postmenopausal women: single nulliparous postmenopausal women have a lower SHBG than married nulliparous postmenopausal women. It is possible that SHBG is unrelated to parity, but that the subset of nulliparous women who are single are characterized by a lower than average SHBG. More data are required to resolve this issue.

The unadjusted values for SHBG show a marked increase with increasing age at menarche in premenopausal women, and a weak trend in the same direction in postmenopausal women. Adjusting these values for QI reduces the magnitude of the relationships. This is because there is a negative correlation between age at menarche and QI in both premenopausal and postmenopausal women. The existence of this relationship, which has been reported before (Garn *et al.*, 1986), provides additional evidence that there may be long term physiological characteristics which are correlated with age at menarche.

Our study suggests that in older premenopausal women (age 34+), SHBG is positively related to age at menarche. In the study by Apter *et al.* (1984) of factors affecting SHBG, the average SHBG concentration at 10.0 to 15.9 years of age was lower in girls with menarche before 13 than in girls with a later menarche, and the early pubertal decrease in SHBG (see Lindstedt *et al.*, 1985) began sooner in girls with relatively early menarche. However, by 16 years of age the difference in SHBG was only 4%. The results of Apter *et al.* were not adjusted for weight, and the comparable figure from our data for premenopausal women (median age 41) is 9% for unadjusted values, and 6% after adjusting for QI, parity (0, 1+), marital status and day of cycle.

MacMahon *et al.* (1982) reported another possible long term difference between women with early or late menarche. They found a significant inverse correlation between age at menarche and urinary oestrogens in women aged 15–19 years. Women aged 30–39 showed a similar but weaker and non-significant correlation. The authors interpreted their results as suggesting that women aged 30–39 who had early menarche still tend to have high oestrogen production, at least in the follicular phase. Our results suggest that, in addition, women who underwent early menarche may have more bioavailable $E_2$ because of the lower SHBG concentrations.

Two recent studies (Dowsett *et al.*, 1985; Plymate *et al.*, 1985) reported increases in SHBG in the luteal phase of the cycle of 15% and 20% respectively. We found that SHBG was about 9% higher in the first 12 days of the luteal phase than in the follicular phase, but then dropped by about 14% in the last 2 days of the cycle. These results appear to be compatible with those of Dowsett *et al.* and Plymate *et al.*: the smaller luteal phase increase found in our study is probably explained by the additional variation due to our study being cross-sectional rather than longitudinal and due to our assumption of a constant 14 day luteal phase in contrast to the biochemical detection of ovulation used in the other studies.

Our observations on the relationship between SHBG and cigarette smoking must be treated cautiously because of the small number of smokers on which they are based. In postmenopausal women we found that SHBG was 5% higher in smokers than in non-smokers, but this was not statistically significant, in agreement with the results of Lapidus *et al.* (1986). In premenopausal women we found that SHBG was 13% higher in smokers than in non-smokers. We have been unable to find another report of SHBG and smoking in premenopausal women. We did not use smoking category as a covariate in analysis of other variables because we only had data on smoking for 28% of the subjects; repeating the analyses in the subset of subjects for whom smoking data were available did not suggest that smoking confounded the other relationships described. We are currently gathering more data on smoking in this population.

We have observed the well established relationship between SHBG and QI and found a correlation between SHBG and age at menarche in premenopausal women. The relationship between parity and SHBG is still unclear. Obesity (in postmenopausal women), early age at menarche, and nulliparity are all risk factors for breast cancer (Pike & Ross, 1984). These factors may affect risk partly through their association with SHBG.

### References

ANDERSON, D.C. (1974). Sex-hormone-binding globulin. *Clin. Endocrinol.*, **3**, 69.

APTER, D., BOLTON, N.J., HAMMOND, G.L. & VIHKO, R. (1984). Serum sex hormone-binding globulin during puberty in girls and in different types of adolescent menstrual cycles. *Acta Endocrinol.*, **107**, 413.

BERNSTEIN, L., PIKE, M.C., ROSS, R.K., JUDD, H.L., BROWN, J.B. & HENDERSON, B.E. (1985). Estrogen and sex hormone-binding globulin levels in nulliparous and parous women. *J. Natl Cancer Inst.*, **74**, 741.

BERNSTEIN, L., DEPUE, R.H., ROSS, R.K., JUDD, H.L., PIKE, M.C. & HENDERSON, B.E. (1986). Higher maternal levels of free estradiol in first compared to second pregnancy: Early gestational differences. *J. Natl Cancer Inst.*, **76**, 1035.

CHAKRAVARTI, S., COLLINS, W.P., FORECAST, J.D., NEWTON, J.R., ORAM, D.H. & STUDD, J.W.W. (1976). Hormonal profiles after the menopause. *Br. Med. J.*, **2**, 784.

CLAIR, P., CLAUSTRAT, B., JORDAN, D., DECHAUD, H. & SASSOLAS, G. (1985). Daily variations of plasma sex hormone-binding globulin binding capacity, testosterone and luteinizing hormone concentrations in healthy rested adult males. *Hormone Res.*, **21**, 220.

DE MOOR, P. & JOOSSENS, J.V. (1970). An inverse relation between body weight and the activity of the steroid binding beta-globulin in human plasma. *Steroidologia*, **1**, 129.

DOWSETT, M., ATTREE, S.L., VIRDEE, S.S. & JEFFCOATE, S.L. (1985). Oestrogen-related changes in sex hormone binding globulin levels during normal and gonadotrophin-stimulated menstrual cycles. *Clin. Endocrinol.*, **23**, 303.

ESTOUR, B., PUGEAT, M., LANG, F., DECHAUD, H., PELLET, J. & ROUSSET, H. (1986). Sex hormone binding globulin in women with anorexia nervosa. *Clin. Endocrinol.*, **24**, 571.

GARN, S.M., LAVELLE, M., ROSENBERG, K.R. & HAWTHORNE, V.M. (1986). Maturational timing as a factor in female fatness and obesity. *Am. J. Clin. Nutr.*, **43**, 879.

HAMMOND, G.L., LANGLEY, M.S. & ROBINSON, P.A. (1985). A liquid-phase immunoradiometric assay for human sex-hormone-binding globulin. *J. Steroid Biochem.*, **23**, 451.

HENDERSON, B.E., ROSS, R.K., PIKE, M.C. & CASAGRANDE, J.T. (1982). Endogenous hormones as a major factor in human cancer. *Cancer Res.*, **42**, 3232.

KOPELMAN, P.G., PILKINGTON, T.R.E., WHITE, N. & JEFFCOATE, S.L. (1980). Abnormal sex steroid secretion and binding in massively obese women. *Clin. Endocrinol.*, **12**, 363.

KUOPPASALMI, K. (1980). Plasma testosterone and sex-hormone-binding globulin capacity in physical exercise. *Scand. J. Clin. Lab. Invest.*, **40**, 411.

LAPIDUS, L., LINDSTEDT, G., LUNDBERG, P.-A., BENGTSSON, C. & GREDMARK, T. (1986). Concentrations of sex-hormone binding globulin and corticosteroid binding globulin in serum in relation to cardiovascular risk factors and to 12-year incidence of cardiovascular disease and overall mortality in postmenopausal women. *Clin. Chem.*, **32**, 146.

LINDSTEDT, G., LUNDBERG, P.-A., HAMMOND, G.L. & VIHKO, R. (1985). Sex hormone-binding globulin – still many questions. *Scand. J. Lab. Invest.*, **45**, 1.

MACMAHON, B., TRICHOPOULOS, D., BROWN, J. & 8 others (1982). Age at menarche, urine estrogens and breast cancer risk. *Int. J. Cancer*, **30**, 427.

MARUYAMA, Y., AOKI, N., SUZUKI, Y., SINOHARA, H. & YAMAMOTO, T. (1984). Variation with age in the levels of sex-steroid-binding plasma protein as determined by radio-immunoassay. *Acta Endocrinol.*, **106**, 428.

MOORE, J.W., CLARK, G.M.G., HOARE, S.A. & 5 others (1986). Binding of oestradiol to blood proteins and aetiology of breast cancer. *Int. J. Cancer*, **38**, 625.

PARTRIDGE, W.M. (1981). Transport of protein-bound hormones into tissues *in vivo*. *Endocr. Rev.*, **2**, 103.

PIKE, M.C. & ROSS, R.K. (1984). Breast cancer. *Br. Med. Bull.*, **40**, 351.

PLYMATE, S.R., MOORE, D.E., CHENG, C.Y., BARDIN, C.W., SOUTHWORTH, M.B. & LEVINSKI, M.J. (1985). Sex hormone-binding globulin changes during the menstrual cycle. *J. Clin. Endocrinol. Metab.*, **61**, 993.

SIITERI, P.K., HAMMOND, G.L. & NISKER, J.A. (1981). Increased availability of serum estrogens in breast cancer: A new hypothesis. In *Hormones and Breast Cancer*, Pike, M.C. *et al.* (eds) p. 87. Cold Spring Harbor Laboratory: New York.